# Exercise and the kidneys: How does renal blood flow behave when measured during exercise?

**DOI:** 10.14814/phy2.15485

**Published:** 2022-10-06

**Authors:** Marcos Paulo Rocha, Lasse Gliemann

**Affiliations:** ^1^ The August Krogh Section for Human Physiology, Department of Nutrition, Exercise and Sports University of Copenhagen Copenhagen Denmark

## COMMENT LETTER

There is compelling scientific evidence for the benefits of regular exercise practice for sedentary, aged, and patient groups as a tool for preventing or improving cardiovascular diseases like hypertension, one of the leading causes of chronic kidney disease (CKD) development (Franczyk et al., 2017; Pedersen & Saltin, 2015). In the long term, untreated high blood pressure cause elevated intraglomerular pressure impacting glomerular filtration (Franczyk et al., 2017). For an adequate prescription of exercise program routines to prevent and improve CKD, aspects like exercise modality, intensity, and volume, must be considered, regardless of the target population. One important thing to consider is that a single bout of exercise increases sympathetic nervous system activation and causes the release of the sympathetic neurotransmitter norepinephrine (NE) in the whole body. The circulating NE will bind the alfa‐adrenergic receptors in the blood vessels, causing a vasoconstriction response. Locally, in the contracting muscles, however, the mechanism of “functional sympatholysis” overrules the local sympathetic activation, permitting an increase in blood flow to meet the increased metabolic demand (Fisher et al., 2015).

While the active muscle escapes the augmented sympathetic neural outflow, renal function and hemodynamics will see pronounced effects. Exercise‐induced sympathetic activity will induce vasoconstriction of afferent arterioles reducing the renal blood flow (DiBona, 2005). The drop in the blood flow to the kidney during exercise has been investigated through various techniques, including doppler ultrasound, in healthy and cardiovascular disease patient groups, all showing reduced renal blood flow response during exercise (Endo et al., 2008; Momen et al., 2006; Tidgren et al., 1991). However, an exaggerated reduction in renal blood flow is found in cardiovascular diseases such as heart failure patients compared to control healthy groups performing exercise (Drew et al., 2013; Middlekauff et al., 2000). In the long term, this could develop into more severe clinical consequences.

However, in the recently published issue by Kawakami et al. entitled “The moderate‐intensity continuous exercise maintains renal blood flow and does not impair the renal function,” the authors reported no change in renal blood flow in response to exercise in healthy middle‐aged men (Kawakami et al., 2022). Kawakami's findings are intriguing and challenge the current literature regarding blood flow response in the renal artery during exercise. However, the authors did not measure the blood flow during exercise but immediately after the termination of exercise. In addition, the authors present a decrease in the cross‐sectional area of the renal artery 30 min post‐exercise. Thus, the question remains, what happens with the renal blood flow during exercise? It would be interesting and a piece of valuable information if the authors had examined the behavior of renal blood flow during the 30 min of exercise. This information would complement the current picture of the renal blood flow before, during, and immediately after exercise.

In a previous study, Endo et al. continuously measured renal blood flow before, during, and after a dynamic exercise and showed a decrease in renal blood flow during exercise, which rapidly returned to baseline levels (within 40 s) after exercise cessation (Endo et al., 2008). Endo et al. findings make it difficult to consider the immediate period after exercise to represent what happens during exercise. The time spent between concluding the exercise bout, visualizing the artery, and recording the blood flow response may influence the interpretation of the current data by Kawakami et al. Thus, Kawakami's findings represent the blood flow response in the early recovery phase after 30 min of exercise and not renal blood flow *during* exercise.

We then argue that the presented data does not support the conclusion when affirming that renal blood flow is not changed during moderate exercise. Instead, the data shows that renal blood flow in the recovery phase immediately after moderate intensity exercise is not different from resting conditions. Conflicting results between studies are not an issue and are quite common in the literature. However, Kawakami's findings of post‐exercise blood flow should be carefully interpreted and should not be confused with renal hemodynamics during exercise.
